# Effects of hydrogen peroxide on diazepam and xylazine sedation in chicks

**DOI:** 10.2478/v10102-012-0030-5

**Published:** 2012-12

**Authors:** Yaareb J. Mousa, Fouad K. Mohammad

**Affiliations:** Department of Physiology, Biochemistry and Pharmacology, College of Veterinary Medicine, University of Mosul, Mosul, Iraq

**Keywords:** oxidative stress, sedation, diazepam, xylazine, H_2_O_2_

## Abstract

Oxidative stress may cause various neuronal dysfunctions and modulate responses to many centrally acting drugs. This study examines the effects of oxidative stress produced by hydrogen peroxide (H_2_O_2_) on sedation induced by diazepam or xylazine as assessed in 7–14 day-old chicks. Day-old chicks were provided with either plane tap water (control group) or H_2_O_2_ in tap water as 0.5% v/v drinking solution for two weeks in order to produce oxidative stress. Spectrophotometric methods were used to determine glutathione and malondialdehyde concentrations in plasma and whole brain. Drug-induced sedation in the chicks was assessed by monitoring the occurrence of signs of sedation manifested as drooping of the head, closed eyelids, reduced motility or immotility, decreased distress calls, and recumbency. The latency to onset of sedation and its duration were also recorded. H_2_O_2_ treatment for two weeks significantly decreased glutathione and increased malondialdehyde concentrations in plasma and whole brain of the chicks on days 7, 10 and 14 as compared with respective age-matched control groups. H_2_O_2_ decreased the median effective doses of diazepam and xylazine for the induction of sedation in chicks by 46% and 63%, respectively. Injection of diazepam at 2.5, 5 and 10 mg/kg, i.m. or xylazine at 2, 4 and 8 mg/kg, i.m. induced sedation in both control and H_2_O_2_-treated chicks in a dose dependent manner, manifested by the above given signs of sedation. H_2_O_2_ significantly decreased the latency to onset of sedation in chicks treated with diazepam at 5 and 10 mg/kg, increased the duration of sedation and prolonged the total recovery time in comparison with respective non-stressed control chicks. A similar trend occurred with xylazine in the H_2_O_2_-treated chicks, though the differences from control counterparts did not attain the statistical significance, except for the recovery time of the lowest dose of the drug. The data suggest that H_2_O_2_-induced oxidative stress sensitizes the chicks to the depressant action of the sedatives diazepam and xylazine. Further studies are needed to examine the potential role of oxidative stress in modulating the actions of therapeutic agents on the brain.

## Introduction

There is ample evidence indicating that reactive oxygen species are involved in various neuronal disorders and diseases (Aksenova *et al.*, 2005; Kunwar & Priyadarsini, 2011). Oxidative stress (OS) may cause various central nervous system (CNS) dysfunctions (Aksenova *et al.*, 2005; Sayre *et al.*, 2008; Ghosh *et al.*, 2011). Stressful conditions involving the brain may also modulate the response to many centrally acting therapeutic agents (Watanabe *et al.*, 1996; Aksenova *et al.*, 2005). Hydrogen peroxide (H_2_O_2_) is used experimentally in vivo to induce OS in various laboratory animal species (Desagher *et al.*, 1996; Servitja *et al.*, 2000; Celik & Ozkaya, 2002, Ahmed, 2010; Al-Baggou *et al.*, 2011) and in vitro for experimental models of OS in cells and tissues (Frantseva *et al.*, 1998; Aksenova *et al.*, 2005; Gonzalez *et al.*, 2006). H_2_O_2_-induced OS was reported to modify the response of rabbits to ketamine-detomidine anesthesia (Wohaieb *et al.*, 1994) and that of rats to pentobarbital anesthesia (Mohammad *et al.*, 1999). A recent study also reported that H_2_O_2_ potentiated the anticholinesterase poisoning induced by the organophosphate insecticides dichlorvos and diazinon in chicks (Al-Baggou *et al.*, 2011).

The neurotoxic effects of H_2_O_2_, through production of free radicals, might be related to lipid peroxidation in the CNS (Piantadosi & Tatro, 1990; Watt *et al.*, 2004) and elevation of intracellular calcium (Hinshaw *et al.*, 1993; Gonzalez *et al.*, 2006). The latter effect could be related to activation of the non-selective cation channel of the cell (Smith *et al.*, 2003). Neurotransmitters, such as glutamate and GABA, could also be the target of H_2_O_2_ in the brain (Aksenova *et al.*, 2005; Gonzalez *et al.*, 2006). In this context, the response of the brain to centrally acting therapeutic agents is expected to be modified. Hence, the purpose of the present study was to examine the effects of OS induced by H_2_O_2_ on diazepam- and xylazine-induced sedation in chicks. Both drugs are centrally acting sedatives in animals, including avian species (Crowell-Davis & Murray, 2006; Rock, 2007).

## Materials and methods

### Animals

Day-old Cobb broiler chicks of both sexes (body weight 40–90 g) were used. They were maintained in a room with a temperature of 32–35 °C, constant lighting and wood shavings as floor litter. The chicks had free access to drinking water and feed throughout the experiment. All experiments complied with our institutional regulations addressing animal use, and the chicks received proper attention and humane care. The Scientific Committee of the College of Veterinary Medicine at the University of Mosul reviewed and approved the protocol of this study.

### Induction of OS

Day-old chicks were either provided with plane tap water (control group) or H_2_O_2_ (Thomas Baker Chemical Ltd., U.K.) in tap water as 0.5% v/v drinking solution for two weeks in order to produce OS as reported before (Mohammad *et al.*, 1999; Ahmed, 2010). We changed the drinking water and supplied it freshly to the chicks on each day. On treatment days 7, 10 and 14, blood samples were obtained from the chicks (8/group) by jugular vein bleeding into heparinized test tubes (Stevens & Ridgway, 1966). Thereafter, the chicks were euthanized by cervical dislocation to obtain the whole brain. Blood samples were centrifuged at 10 000 rpm for 15 minutes to obtain plasma. Plasma and whole brain samples were kept at –20 °C pending analysis within one week. Spectrophotometric methods were used to determine glutathione concentration in the brain by a modified Ellman method (Ellman, 1959; James *et al.*, 1982) and malondialdehyde concentration was determined by the method of Ohkawa *et al.* (1979). We dissolved diazepam (kindly donated by the State Company for Drugs and Medical Appliances-Ninevah, Iraq) in warm propylene glycol, and further diluted xylazine (2%, Alfasan Co., Holland) in normal saline solution to obtain the desired concentrations of the drugs for injection at a volume of 5 ml/kg body weight given intramuscularly (i.m.).

### Effect of H_2_O_2_ on sedation induced by diazepam and xylazine

The up-and-down method (Dixon, 1980) was used to determine the median effective doses (ED50s) of diazepam and xylazine for the induction of sedation in chicks given tap water (control) or H_2_O_2_ as described above. The age of the chicks assessed in this experiment ranged between 7–14 days. The initial dose of diazepam or xylazine was at 10 mg/kg, i.m. After the injection of each drug, we monitored the chicks for the occurrence of sedation manifested as drooping of the head, closed eyelids, reduced motility or immotility, decreased distress calls, and recumbency (Al-Zubaidy & Mohammad, 2005). In another experiment, we monitored the dose-response sedative effects of diazepam and xylazine in the two groups of H_2_O_2_-stressed and non-stressed chicks. The chicks (8/dose group) were treated i.m. with diazepam at 2.5, 5 and 10 mg/kg or xylazine at 2, 4 and 8 mg/kg. After injection of each drug, the chicks were individually monitored to record the onset of sedation (drooping of the head) and its duration, as well as the total recovery time as described previously (Al-Zubaidy & Mohammad, 2005). Recovery time was the time from the onset of sedation until the chick moved freely. Chicks treated with the vehicle only (propylene glycol or normal saline at 5 ml/kg, i.m.) were also included in all the experiments.

### Statistics

The data were statistically analyzed by analysis of variance followed by the least significant difference test (Petrie & Watson, 1999). The level of significance was at *p<*0.05.

## Results

H_2_O_2_ treatment in drinking water for two weeks significantly decreased glutathione and increased malondialdehyde concentrations in plasma and whole brain of the chicks on days 7, 10 and 14 as compared with respective age-matched control groups (Tables 1 and 2). The ED50s of diazepam and xylazine for the induction of sedation in control chicks, as determined by the up-and-down method, were 6.5 and 4.8 mg/kg, i.m., respectively. In the H_2_O_2_-treated chicks the sedative ED50s of the drugs decreased by 46% and 63% to 3.5 and 1.8 mg/kg, i.m., respectively.

**Table 1:** Glutathione concentrations in plasma and whole brain of chicks treated with hydrogen peroxide (0.5%) in drinking water for two weeks.

	Age (days)		
Groups	7	10	14
**Plasma (µmol/ml)**			
Control	0.213±0.032	0.261±0.022	0.269±0.023
H_2_O_2_	0.062±0.007*	0.137±0.015* a	0.143±0.010* a
**Whole brain (µmol/g)**			
Control	0.477±0.076	0.571±0.039	0.911±0.100^a^ ^b^
H_2_O_2_	0.187±0.025*	0.352±0.036* a	0.458±0.048* a

Values are mean ± SE for 8 chicks/group

Hydrogen peroxide was added to drinking water of one-day-old chicks and continued for 14 days.

* Significantly different from the respective control value, *p<*0.05.

a Significantly different from the respective 7-day-old group, *p<*0.05.

b Significantly different from the respective 10-day-old group, *p<*0.05.

**Table 2:** Malondialdehyde concentrations in plasma and whole brain of chicks treated with hydrogen peroxide (0.5%) in drinking water for two weeks.

	Age (days)		
Groups	7	10	14
**Plasma (nmol/ml)**			
Control	3.3±0.59	3.7±0.63	8.5±2.01^a^ ^b^
H_2_O_2_	13.9±0.64*	15.5±3.14*	26.5±2.89* a b
**Whole brain (nmol/g)**			
Control	123.9±7.75	138.5±7.61	125.1±6.37
H_2_O_2_	182.1±4.5*	171.8±12.08*	163.9±7.45*

Values are mean ± SE for 8 chicks/group

Hydrogen peroxide was added to drinking water of one–day- old chicks and continued for 14 days.

* Significantly different from the respective control value, *p<*0.05.

a Significantly different from the respective 7-day-old group, *p<*0.05.

b Significantly different from the respective 10-day-old group, *p<*0.05.

Injections of diazepam and xylazine induced sedation in the control chicks in a dose dependent manner within 2.6 to 5.1 and 1.6 to 2.5 min, respectively (Tables 3 and 4). The chicks manifested signs of sedation characterized by drooping of the head and wings, closed eyelids, reduced motility or immotility, recumbency and decreased distress calls. The duration of sedation ranged between 55 to 92.3 min for diazepam and between 23.1 to 46.9 min for xylazine, with total recovery times of 82.4 to 115.3 and 27.5 to 65.9 min, respectively (Tables 3 and 4). None of the signs of sedation appeared in the vehicle-treated chicks. H_2_O_2_ significantly decreased the latency to onset of sedation in chicks treated with diazepam at 5 and 10 mg/kg, increased the duration of sedation and prolonged the total recovery time in comparison with respective non-stressed control chicks (Table 3). We observed a similar trend with xylazine in the H_2_O_2_-treated chicks, though the differences with control counterparts did not attain the statistical significance, except at the recovery time of the lowest dose of the drug (Table 4).

**Table 3:** Sedative effect of diazepam in chicks treated with hydrogen peroxide (0.5%) in drinking water for two weeks.

Diazepam (mg/kg, i.m.)	Duration of sedation (min)	Latency to onset of sedation (min)	Recovery time (min)
**Control (tap water)**			
2.5	55.0±4.74	5.1±0.55	82.4±3.91
5	74.6±4.44^a^	4.1±0.77	94.4±6.24
10	92.3±6.47^a^ ^b^	2.6±0.38^a^	115.3±6.64^a^ ^b^
**Hydrogen peroxide (0.5% in water)**			
2.5	44.5±6.32	4.4±0.75	79.3±9.70
5	94.9±6.48* a	2.5±0.19* a	121.9±4.02* a
10	124.9±6.72* a b	1.3±0.16* a	203.5±7.45* a b

Values are mean ± SE for 8 chicks/group

Hydrogen peroxide was added to drinking water of one- day old chicks and continued for 14 days.

* Significantly different from the respective control value, *p<*0.05.

a Significantly different from the respective 2.5 mg/kg dose group of diazepam within the same water treatment regimen, *p<*0.05.

b Significantly different from the respective 5 mg/kg dose group of diazepam within the same water treatment regimen, *p<*0.05.

**Table 4:** Sedative effect of xylazine in chicks treated with hydrogen peroxide (0.5%) in drinking water for two weeks.

Xylazine (mg/kg, i.m.)	Duration of sedation (min)	Latency to onset of sedation (min)	Recovery time (min)
**Control (tap water)**			
2	23.1±1.00	2.5±0.33	27.5±1.39
4	34.1±2.62^a^	1.9±0.23	45.1±4.70^a^
8	46.9±4.04^a^ ^b^	1.6±0.74^a^	65.9±8.64^a^ ^b^
**Hydrogen peroxide (0.5% in water)**			
2	27.5±3.05	1.8±0.31	37.1±4.33*
4	39.0±2.54^a^	1.5±0.19	54.3±4.68^a^
8	52.1±3.10^a^ ^b^	1.3±0.25	75.9±7.8^a^ ^b^

Values are mean ± SE for 8 chicks/group

Hydrogen peroxide was added to drinking water of one-day-old chicks and continued for 14 days.

* Significantly different from the respective control value, *p<*0.05.

a Significantly different from the respective 2 mg/kg dose group of xylazine within the same water treatment regimen, *p<*0.05.

b Significantly different from the respective 4 mg/kg dose group of xylazine within the same water treatment regimen, *p<*0.05.

## Discussion

The decrease in glutathione and increase in malondialdehyde concentrations in plasma and whole brain of the chicks after oral exposure to H_2_O_2_ suggest that the treatment in drinking water induced OS in the chicks, including their CNS. Glutathione and malondialdehyde are indirect markers of OS in blood and tissues (Janero, 1990; Kohen & Nyska, 2002; Aksenova *et al.*, 2005; Santi *et al.*, 2011). Glutathione is an oxygen radical scavenger, whereas malondialdehyde is the by-product of membrane peroxidation that results in tissue damage and further indicates reduced effectiveness of the mechanisms of antioxidants in protecting the tissue from oxidative damage (Sies, 1999; Karadeniz *et al.*, 2007; Limon-Pacheco *et al.*, 2007). The OS appeared unequivocally on days 7, 10 and 14 of exposure. These results are in accordance with the reported findings that H_2_O_2_ oral exposure could be used as a model for the induction of OS in chickens (Ahmed, 2010; Al-Baggou *et al.*, 2011) as well as in rodent species (Desagher *et al.*, 1996; Servitja *et al.*, 2000; Celik & Ozkaya, 2002). The CNS is particularly susceptible to OS-induced neurotoxicity (Sayre *et al.*, 2008). H_2_O_2_ differentially accumulates in various brain regions (Piantadosi and Tatro, 1990), sets free oxygen radicals that might induce cellular and DNA damage (Gutteridge, 1995; Lee and Jeong, 2007; Xiao-yan *et al.*, 2010; Kunwar & Priyadarsini, 2011). It is also possible that neuronal function is altered as a result of lipid peroxidation, which changes membrane fluidity and damages the protein components of neurons (Kunwar & Priyadarsini, 2011). Aldehydes and ketones are toxic products of protein oxidation (Kunwar & Priyadarsini, 2011). Furthermore, in vitro experiments showed that oxidative stress caused phospholipid damage in brain slices and astrocytes (Servitja *et al.*, 2000). Oxidative stress as a form of neurotoxic condition (Sayre *et al.*, 2008) was reported to severely damage neurotransmitter signaling systems and modulate enzyme activities, e.g. cholinesterases in the CNS, which might in turn produce an additional burden on neuronal functions (Schallreuter *et al.*, 2004; de Jongh *et al.*, 2007).

Chicks exposed orally to H_2_O_2_ appeared to be more susceptible to the sedative effects of diazepam and xylazine. This was evident by the decreases in the ED50s of both drugs for the induction of sedation. Further, H_2_O_2_-stressed chicks showed prolonged sedation induced by the drugs. Diazepam and xylazine are CNS active drugs with different mechanisms of action (Crowell-Davis & Murray, 2006; Rock, 2007). Diazepam action is mediated by potentiation of GABA-ergic inhibition in the CNS, whereas that of xylazine is mediated by activation of alpha2-adrencoceptors resulting in reduced catecholamine release and turnover (Greene & Thurmon, 1988; Crowell-Davis & Murray, 2006). Corroborating our findings, H_2_O_2_ treatment in drinking water was also found to sensitize rats and rabbits to the sedative and anesthetic actions of pentobarbital and deteomidine-ketamine, respectively (Wohaeib *et al.*, 1994; Mohammad *et al.*, 1999). Other non-chemical stressful conditions, such as restraining and swimming, were reported to alter the pharmacological responses of mice, rats or chicks to diazepam (Marin & Arce, 1996; Kalman *et al.*, 1997; Motohashi *et al*, 2008). Furthermore, H_2_O_2_ potentiated the toxicity of organophosphate insecticides acting mainly through a central mechanism in chicks, irrespective of the extent of cholinesterase inhibition (Al-Baggou *et al.*, 2011). In this context, the data of the present study suggest that H_2_O_2_-induced OS sensitizes the chicks (as a model) to the depressant actions of the sedative drugs diazepam and xylazine. Further studies are needed to get insight into the potential role of OS in modulating the actions of therapeutic agents on the CNS.
